# Serum cAMP levels are increased in patients with asthma

**DOI:** 10.1172/JCI186937

**Published:** 2025-01-07

**Authors:** Steven S. An, Gaoyuan Cao, Kwangmi Ahn, Jordan Lee, Dae Young Jung, Loren Denlinger, John Fahy, Elliot Israel, Wendy Moore, Brenda Phillips, David Mauger, Sally Wenzel, Reynold A. Panettieri

**Affiliations:** 1 Rutgers Institute for Translational Medicine and Science, New Brunswick, New Jersey, USA.; 2 National Human Genome Research Institute, Bethesda, Maryland, USA.; 3 Division of Allergy, Pulmonary and Critical Care Medicine, University of Wisconsin School of Medicine and Public Health, Madison, Wisconsin, USA.; 4 Cardiovascular Research Institute, UCSF, San Francisco, California, USA.; 5 Brigham & Women’s Hospital, Harvard Medical School, Boston, Massachusetts, USA.; 6 Wake Forest School of Medicine, Winston-Salem, North Carolina, USA.; 7 Pennsylvania State University, Hershey, Pennsylvania, USA.; 8 Department of Environmental and Occupational Health, University of Pittsburgh School of Public Health, Pittsburgh, Pennsylvania, USA.

**Keywords:** Cell biology, Pulmonology, Asthma, Cyclic nucleotides, G protein&ndash;coupled receptors

**To the Editor:** As a cornerstone in the treatment of asthma, β2-agonists prevent or reverse the shortening of human airway smooth muscle (HASM), the pivotal cell regulating bronchomotor tone. β2-Agonists act upon β2-adrenoceptor (β2AR) — the cognate Gs-coupled G protein-coupled receptor (Gs-GPCR) expressed on HASM — and activate adenylyl cyclase, which generates 3′,5′-cyclic adenosine monophosphate (cAMP) (1). Increased intracellular cAMP levels ([cAMP]i) consequently stimulate protein kinase A that in turn modulates multiple downstream targets to promote HASM relaxation and reverse airflow obstruction (2).

Classically, the signal transduction evoked by β2ARs is short lived and multiple mechanisms ensure homeostatic regulation of [cAMP]i, with phosphodiesterase (PDE) degradation of cAMP considered to play a dominant role. Using primary HASM cells in culture as a model, we recently reported that β2AR activation evokes cAMP egress to the extracellular space ([cAMP]e) that is long lived in culture, independent of PDE activity, and mediated by ABCC1 (ATP-binding cassette subfamily C member 1) membrane transporter (3). Inhibition of ABCC1 activity or expression decreases cAMP egress, increases [cAMP]i, and enhances HASM relaxation elicited by structurally diverse agonists acting upon Gs-GPCRs (3). These findings suggest a class effect of Gs-GPCR activation and identify ABCC1 as a previously unrecognized cAMP signal response modifier in HASM. Of note, in a small cohort of patients with and without asthma, we detected increased cAMP levels in the blood of patients with asthma (3).

To further explore the clinical utility of detecting circulating cAMP, we measured cAMP levels in a serum biobank from the Severe Asthma Research Program 3 (SARP-3) (4). For this study, we obtained 87 serum samples of patients with asthma, of which 39 are characterized as “severe” according to European Respiratory Society/American Thoracic Society criteria for asthma severity (Supplemental Tables 1 and 2; supplemental material available online with this article; https://doi.org/10.1172/JCI186937DS1) (5). Since SARP-3 did not have a sufficient serum biobank of healthy controls, we leveraged the database from the Rutgers Corona Cohort (RCC) study (6) and obtained 273 serum samples of the study participants without a known history of asthma or other lung diseases, shown in Supplemental Table 1 (see detailed inclusion and exclusion criteria in Supplemental Methods).

We detected a high variability or a wide spread of cAMP levels in 87 serum samples of patients with asthma (Figure 1A), ranging from 0.291 to 563.9 picomole. In contrast, the range of cAMP levels in 273 serum samples of individuals without asthma was markedly smaller (0 to 27.72 picomole) (Figure 1A). Compared with the nonasthma group (0.520 picomole, median), serum cAMP levels were significantly higher in patients with asthma (6.220 picomole, median) (Supplemental Table 1). To further test the hypothesis that cAMP levels can differentiate asthma severity (severe versus nonsevere) in the SARP-3 samples, we applied linear regression models with age and sex as covariates across clinical groups. There was no significant difference of serum cAMP levels between severe asthma and nonsevere asthma (Supplemental Table 2), but each asthma group showed significantly higher cAMP levels (adjusted P < 0.00001) than the nonasthma group (Figure 1B).

Using the SARP-3 data, we next assessed whether measured serum cAMP levels are associated with any clinical traits of asthma. Specifically, we asked whether serum cAMP levels had associations with (a) asthma endotypes; (b) poor control indicators; and (c) postbronchodilator airflow reversibility. There were no significant differences in cAMP levels among or between groups stratified by eosinophilic or neutrophilic asthma (Supplemental Figure 1) and any of the poor control indicators (Supplemental Figure 2). In addition, we did not detect significant differences in serum cAMP levels with maximum FEV1 reversibility with albuterol (Supplemental Figure 3A). Of note, serum cAMP levels arithmetically increased with the number of inhaled corticosteroids (ICS) puffs and controllers used (Supplemental Figure 1) and, in the nonsevere asthma group, increased with the increases of postbronchodilator lung function (Supplemental Figure 3, B and C). Further studies are necessary to explore the link between serum cAMP levels with (a) bronchodilator or treatment responses by asthma severity; (b) ABCC1 expression and activity in health and disease, including specific cell types of origin; and, (c) whether these physiological outcomes and clinical phenotypes are affected by variations in ABCC1 genotypes in a large cohort of patients with and without asthma.

## Supplementary Material

Supplemental data

Supporting data values

## Figures and Tables

**Figure 1:**
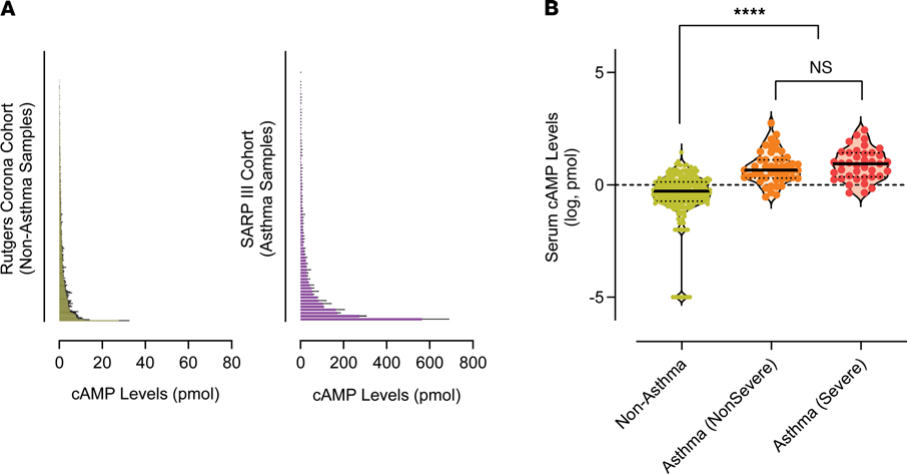
Serum cAMP levels are increased in patients with asthma. (**A**) Histogram of cAMP levels detected in the blood of RCC study participants without a known history of asthma or other lung diseases (*n* = 273) and patients with asthma in the SARP-3 (*n* = 87). For each sample, cAMP levels (pmol per 60 μl serum) were measured in duplicate by using the cAMP-Screen System ELISA kit (Applied Biosystems) and presented as mean ± SD. (**B**) Serum cAMP levels by asthma severity. Asthma severity was defined as “nonsevere” (*n* = 48) and “severe” (*n* = 39) according to European Respiratory Society/American Thoracic Society criteria. Linear regression models were used with age and sex as covariates across clinical groups. To satisfy the normalization assumption necessary for linear regression testing, cAMP levels underwent log transformation and the analysis was conducted using R version 4.4.1.

## Version 1 01/07/2025

In-Press Preview

## Version 2 03/03/2025

Electronic publication
