# Supplementary material for: Serum cAMP levels are increased in patients with asthma
Source: J Clin Invest. 2025 Jan 7;135(5):e186937. doi: 10.1172/JCI186937 (PMC11870724; doi:10.1172/JCI186937)
Supplement: Supplemental data [file jci-135-186937-s027.pdf]

Supplementary Information

Serum cAMP levels are increased in patients with asthma

Steven S. An,[1,‖,*] Gaoyuan Cao,[1,‖] Kwangmi Ahn,[2] Jordan Lee,[1] Dae Young Jung,[1] Loren Denlinger,[3] John Fahy,[4] Elliot Israel,[5] Wendy Moore,[6] Brenda Phillips,[7] David Mauger,[7] Sally Wenzel,[8] and Reynold A. Panettieri, Jr.[1,*]

[1]Rutgers Institute for Translational Medicine and Science, New Brunswick, NJ; [2]National Human Genome Research Institute, Bethesda, MD; [3]Division of Allergy, Pulmonary and Critical Care Medicine, University of Wisconsin School of Medicine and Public Health, Madison, WI; [4]Cardiovascular Research Institute, UCSF, San Francisco, CA; [5]Brigham & Women's Hospital, Harvard Medical School, Boston , MA; [6]Wake Forest School of Medicine, Winston-Salem, NC; [7]Pennsylvania State University, Hershey, PA; [8]Department of Environmental and Occupational Health, University of Pittsburgh School of Public Health, Pittsburgh, PA.

[*]**Correspondence:**
Steven S. An, Ph.D. (sa1510@rbhs.rutgers.edu)
Reynold A. Panettieri, Jr., M.D. (rp856@rbhs.rutgers.edu)
Mailing Address: 89 French Street, New Brunswick, NJ 08901
Phone: 732-235-9132

[‖]**Equal Contribution**

Prelude to the study design and methods: In 1970, Earl W. Sutherland wrote (1), *"I would be willing to predict that scientists will still be studying the cyclic nucleotides 25 years from now, and it seems not unrealistic to hope that somewhere along the line, as a happy by-product of this research, a new or improved method of therapy will become available."* This statement is every bit true today as was then and, for the asthma management, has ushered in new and improved inhaled β_2-agonist bronchodilators. β_2-agonists act upon β_2-adrenoceptors (β_2ARs)–the cognate G_s-coupled G protein-coupled receptor (G_s-GPCR) expressed on the smooth muscle of human bronchi–and activate a canonical adenylyl cyclase which generates 3',5'-cyclic adenosine monophosphate (cAMP) (2–5). Increased intracellular cAMP levels ($[cAMP]_i$) consequently stimulate protein kinase A that in turn phosphorylates myosin light chain kinase and related proteins to evoke human airway smooth muscle (HASM) relaxation and reverse airflow obstruction (6, 7).

Sutherland recognized early three key doctrines on the mechanism of actions of cAMP (2, 3): (i) a short-lived cytosolic signaling molecule [second messenger] involved in a wide range of physiological responses; (ii) a fast binary switch-like action of adenylyl cyclase [synthesis] and phosphodiesterase [termination]; and, (iii) a long-lived excretion into extracellular fluids (i.e., urine, plasma and cerebrospinal fluid)–all of which are tightly controlled to regulate $[cAMP]_i$. What Sutherland was unaware of at the time, and what has been largely ignored by others to date, is the diagnostic value and clinical translation of the cAMP egress. Here, we explored the clinical utility of detecting circulating cAMP in patients with asthma.

Sex as a biological variable: We obtained serum samples of both sexes (male and female) from the participants in the SARP-3 and RCC study (Supplementary Table 1).

Severe Asthma Research Program (SARP)-3 Cohort: SARP-3 is an observational study (started in November 2012) that is enriched in patients with well-characterized severe asthma (8).

Since children may outgrow asthma during adolescence (9), we focused on adult participants (≥ 18 years of age at enrollment) in the SARP-3 and obtained 87 serum samples of patients with asthma (collected on the year 3 annual visit with severity and maximum FEV_1 reversibility categories), of which 39 are characterized as “severe” according to European Respiratory Society / American Thoracic Society criteria for asthma severity (Supplementary Table 1–2) (8-10). Because SARP-3 only collected serum from healthy (non-asthma) subjects at a single visit and did not have a sufficient serum biobank of healthy controls (age- and sex-matched), we leveraged the database from the Rutgers Corona Cohort (RCC) study.

Rutgers Corona Cohort (RCC) Study: In the RCC study (11), from March 24–April 7, 2020, baseline data and serum were collected from healthcare workers (HCW, N=546) and non-healthcare workers (NHCW, N=283), before developing COVID-19. As described previously (11, 12), eligible HCW reported: (i) ≥ 20 h of hospital work weekly; (ii) occupations with regular patient exposure (e.g., residents, fellows, attending physicians, dentists, nurse practitioners, physician assistants, registered nurses, technicians, respiratory therapists, physical therapists); and (iii) regular direct patient contact (≥ 3 patients/shift) expected in the next 3 months. Eligibility criteria for NHCW included: (i) faculty, staff, trainees, or students working at Rutgers ≥ 20 h weekly; and (ii) no patient contact. For both groups, additional eligibility criteria were: (i) ≥ 20 years of age; (ii) not pregnant or breastfeeding; (iii) no urgent care or emergency room visits, hospitalizations, operations, or changes in prescription medicines in the prior 30 days; and (iv) no previously diagnosed SARS-CoV-2 infection or COVID-19. We obtained 273 serum samples of the study participants without a known history of asthma or other lung diseases (Supplementary Table 1).

cAMP assay: We received Visit 6 samples from the SARP-3 (8), which were collected between December 2015 and May 2018 and re-aliquoted into smaller aliquots in Spring–Summer of 2022. Of note, within an hour of collection, the serum was isolated and stored at -80°C; there was one

freeze/thawed cycle before shipping. We received the samples in cryotubes (Fisher Scientific, #02-681-333) with a yellow O-ring screw cap (Fisher Scientific, #02-681-360) with 50 μl of serum in each tube in July–September of 2022. Samples were immediately stored at -80°C before the measurements. In the RCC study (11, 12), the serum was collected in March–April of 2020 and stored at -80°C in 0.75 ml cryotubes from Micronic, Inc.

cAMP-Screen System ELISA kit (catalog number 4412182, Applied Biosystems) was used for human blood serum cAMP quantitation according to the manufacturer's protocol. For this study, we used 50 μl of serum samples from 87 participants in the SARP-3 and 273 participants in the RCC study. For each patient sample, 50 μl of serum was first diluted 1:3 in lysis buffer and mixed with 60 μl cAMP antibody and 30 μl conjugate (provided by Applied Biosystems). Serum-Ab-Conjugate mixtures were loaded in duplicates with standards to 96-well ELISA plates (the final serum volume loaded to each well was 20 μl). The plates were incubated on an Orbital Shaker at 70 rpm for 60 min followed by 5X wash, and 100 μl of the substrate from the ELISA kit was loaded and incubated for 30 min in the dark. Plates were read with CLARIOstar plate reader (BMG LABTECH). cAMP levels are presented as pmol per 60 μl serum.

Statistics: Unless otherwise stated, we used linear regression models with age and sex as covariates across clinical groups. To satisfy the normalization assumption necessary for linear regression testing, cAMP levels underwent log transformation. The analysis was conducted using R version 4.4.1.

Study approval: All subjects provided informed consent at the respective study sites (RCC and SARP-3), and the study was approved by the Rutgers IRB.

Data availability: The authors declare that all data supporting the findings of this study are available within the paper and its Supplementary Information. The source data for the figures and any supporting data are available upon request.

References

1. Sutherland EW. On the biological role of cyclic AMP. *JAMA.* 1970;214(7):1281–1288.
2. Rall TW, et al. The relationship of epinephrine and glucagon to liver phosphorylase. *J Biol Chem.* 1957;224(1):463–475.
3. Sutherland EW, Rall TW. Fractionation and characterization of a cyclic adenine ribonucleotide formed by tissue particles. *J Biol Chem.* 1598;232(2):1077–1091.
4. Limbird LE, Lefkowitz RJ. Resolution of beta-adrenergic receptor binding and adenylate cyclase activity by gel exclusion chromatography. *J Biol Chem.* 1977;252(2):799–802.
5. Ross EM, et al. Reconstitution of hormone-sensitive adenylate cyclase activity with resolved components of the enzyme. *J Biol Chem.* 1978;253(18):6401–6412.
6. Komalavilas P, et al. The small heat shock-related protein, HSP20, is a cAMP-dependent protein kinase substrate that is involved in airway smooth muscle relaxation. *Am J Physiol.* 2008;294(1):L69–L78.
7. Morgan SJ, et al. β-agonist-mediated relaxation of airway smooth muscle is protein kinase A-dependent. *J Biol Chem.* 2014;289(33):23065–23074.
8. Teague WG, et al. Baseline features of the severe asthma research program (SARP III) cohort: differences with age. *J Allergy Clin Immunol Pract.* 2018;6(2):545–554.
9. Ross KR, et al. Severe asthma during childhood and adolescence: A longitudinal study. *J Allergy Clin Immunol.* 2020;145(1):140–146.
10. Chung KF, et al. International European Respiratory Society/American Thoracic Society guidelines on definition, evaluation and treatment of severe asthma. *Eur Respir J.* 2014;43(2):343–373.

11. Barrett ES, et al. Prevalence of SARS-CoV-2 infection in previously undiagnosed health care workers in New Jersey, at the onset of the U.S. COVID-19 pandemic. *BMC Infect Dis.* 2020;20(1):853.
12. Horton DB, et al. Determinants and dynamics of SARS-CoV-2 infection in a diverse population: 6-month evaluation of a prospective cohort study. *J Infect Dis.* 2021;224(8):1345–1356.
13. Flinkman E, et al. Association between blood eosinophils and neutrophils with clinical features in adult-onset asthma. *J Allergy Clin Immunol Pract.* 2023;11(3):811–821.

Supplementary Figure Legends:

Supplementary Figure 1: Serum cAMP levels in the SARP-3 by asthma endotypes. The cutoff values used to define eosinophilic and neutrophilic asthma were 300 cells/μl and 4000 cells/μl blood, respectively (13). cAMP levels are presented as pmol per 60 μl serum.

Supplementary Figure 2: Serum cAMP levels versus poor control indicators of asthma in the SARP-3. (**A**) Asthma Control Test (ACT) Score, number of exacerbations, and unscheduled doctor visit for breathing problems in the past 12 months. (**B**) Number of months inhaled corticosteroid (ICS) used in the last 12 months, number of ICS puffs in a day, current ICS uses over the past 3 months, and oral steroids (a minimum of 3 days) received for asthma exacerbations in the past 12 months. (**C**) Number of controllers used in the past 12 months, number of short-acting β-agonists (SABA) puffs in a day, SABA and long-acting β-agonists (LABA) uses over the past 3 months. cAMP levels are presented as pmol per 60 μl serum.

Supplementary Figure 3: Serum cAMP levels in the SARP-3 by maximum bronchodilator (BD) response. (**A**) Maximum BD reversibility (maximum FEV_1 reversibility, relative difference [(Post-BD – Pre-BD) / Pre-BD]) of the SARP-3 cohort included in the study. Maximum BD responses (FEV_1, FVC, and FEV_1/FVC) of (**B**) nonsevere and (**C**) severe asthma. FEV_1, forced expired volume in 1 second; FVC, forced vital capacity. cAMP levels are presented as pmol per 60 μl serum.

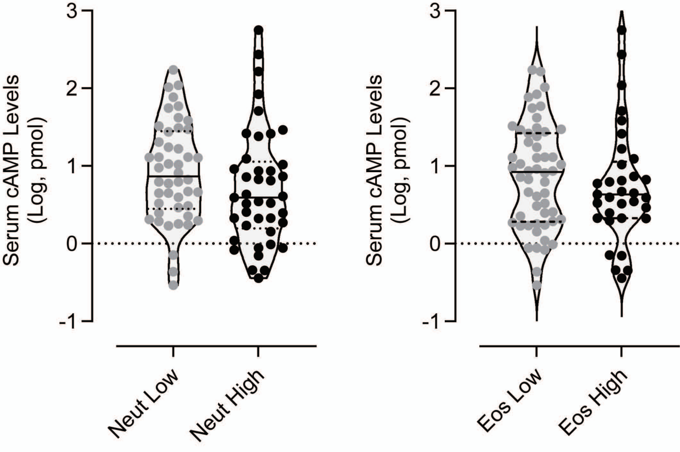

Serum cAMP Levels (Log, pmol)
3
2
1
0
-1
Neut Low
Neut High
Serum cAMP Levels (Log, pmol)
3
2
1
0
-1
Eos Low
Eos High

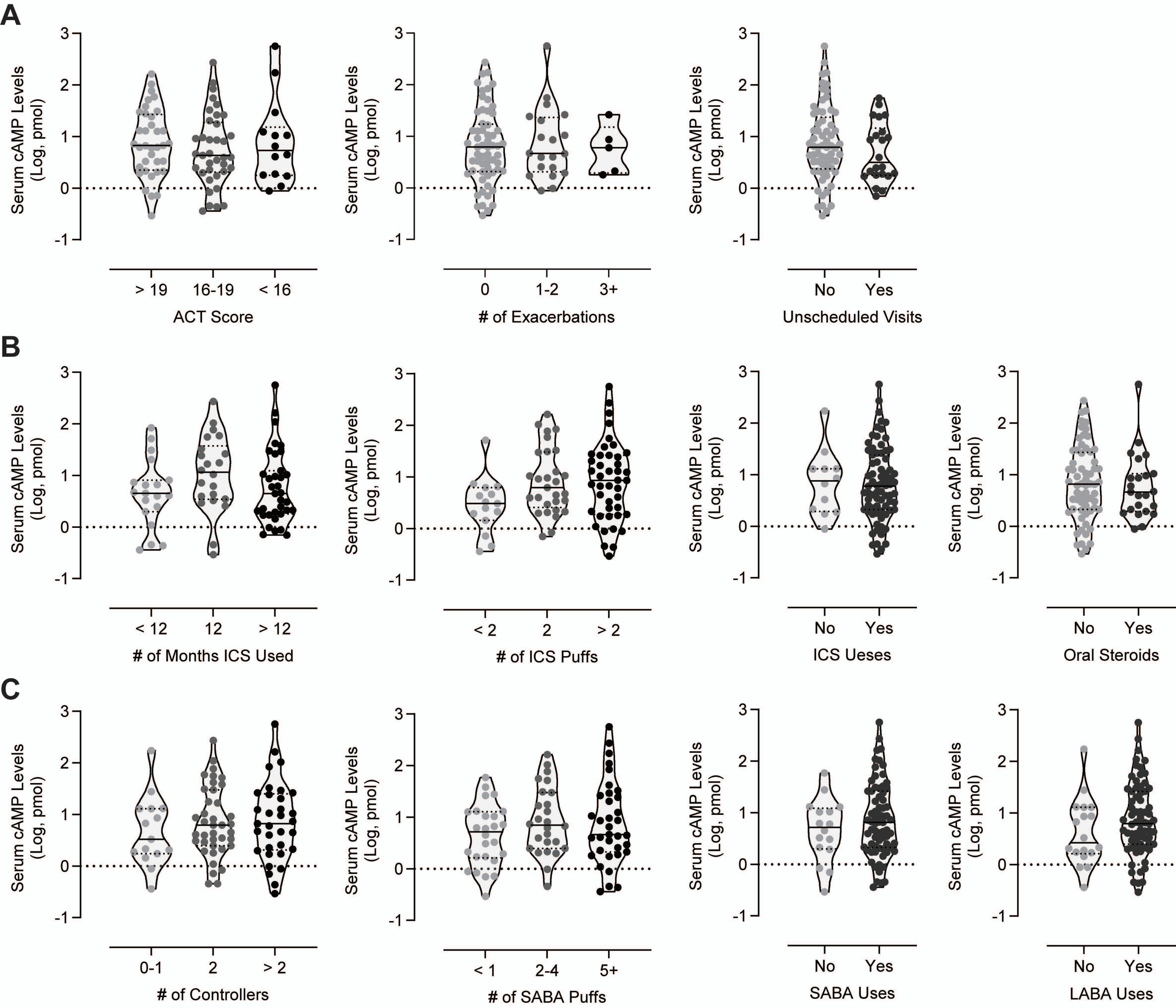

A
Serum cAMP Levels (Log, pmol)
> 19
16-19
< 16
ACT Score
0
1-2
3+
# of Exacerbations
No
Yes
Unscheduled Visits
B
< 12
12
> 12
# of Months ICS Used
< 2
2
> 2
# of ICS Puffs
ICS Ueses
Oral Steroids
C
0-1
2
> 2
# of Controllers
< 1
2-4
5+
# of SABA Puffs
SABA Uses
LABA Uses

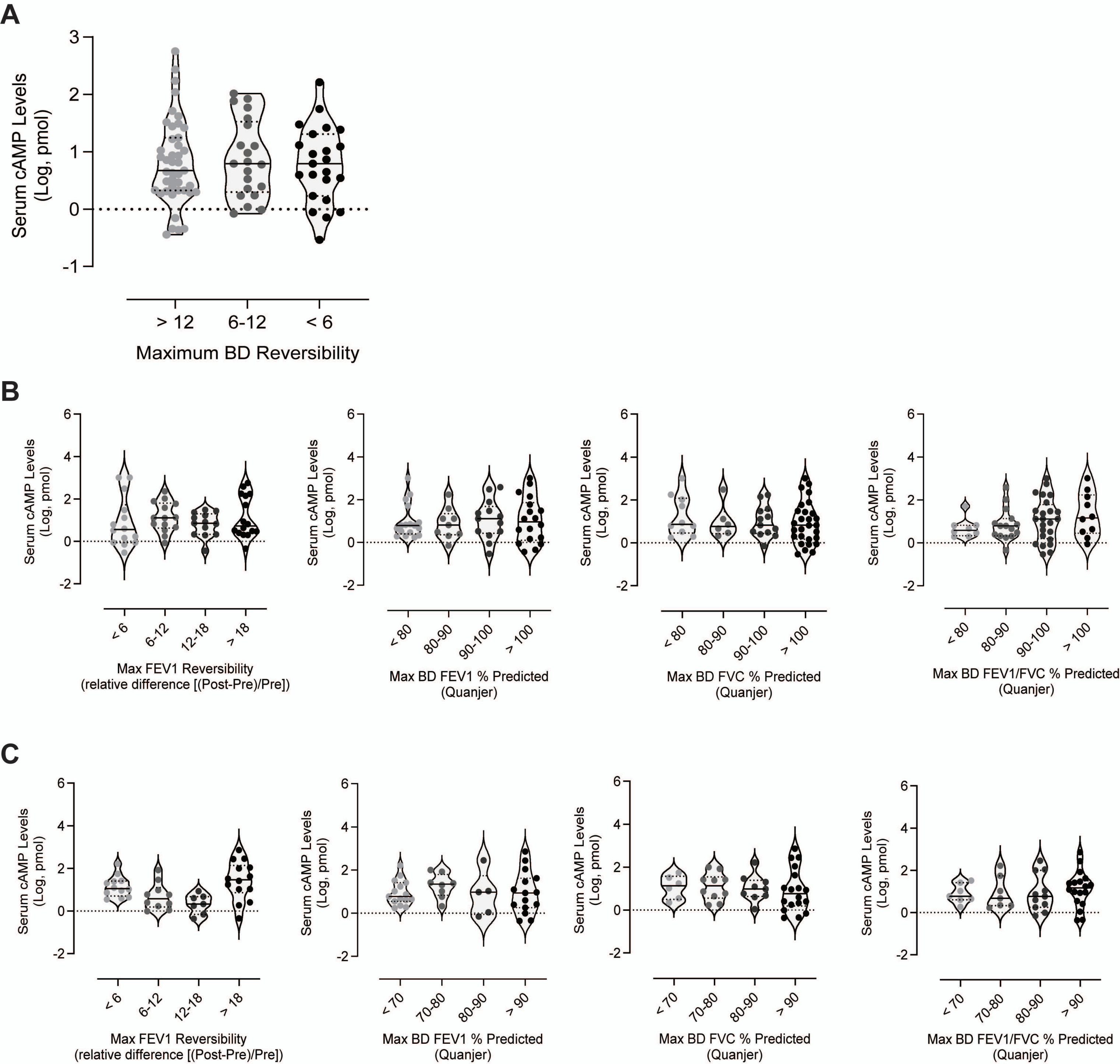
A
Serum cAMP Levels (Log, pmol)
> 12
6-12
< 6
Maximum BD Reversibility
B
Serum cAMP Levels (Log, pmol)
< 6
6-12
12-18
> 18
Max FEV1 Reversibility (relative difference [(Post-Pre)/Pre])
< 80
80-90
90-100
> 100
Max BD FEV1 % Predicted (Quanjer)
Max BD FVC % Predicted (Quanjer)
Max BD FEV1/FVC % Predicted (Quanjer)
C
Serum cAMP Levels (Log, pmol)
Max FEV1 Reversibility (relative difference [(Post-Pre)/Pre])
< 70
70-80
80-90
> 90
Max BD FEV1 % Predicted (Quanjer)
Max BD FVC % Predicted (Quanjer)
Max BD FEV1/FVC % Predicted (Quanjer)

Supplementary Table 1. Key demographic variables and cAMP levels of serum donors from the Rutgers Corona Cohort (RCC) study and the Severe Asthma Research Program (SARP)-3 included in the study

**Serum Donor (no. [%])**	**RCC (No Asthma)**	**SARP-3 (Asthma)**	***P* value**
Age (yr, range)			< 0.0001
20-39	148/273 (54%)	18/87 (21%)	
40-59	108/273 (40%)	35/87 (40%)	
60+	17/273 (6%)	34/87 (39%)	
Sex			0.0319
Male	95/273 (35%)	27/87 (31%)	
Female	178/273 (65%)	60/87 (69%)	
Race			< 0.0005
White	134/273 (49%)	60/87 (69%)	
Black or African American	20/273 (7%)	13/87 (15%)	
Asian	66/273 (24%)	9/87 (10%)	
Hispanic	37/273 (14%)	3/87 (4%)	
Other / Missing	16/273 (6%)	2/87 (2%)	
**Serum cAMP Levels (pmol)**			< 0.001
Minimum	0	0.291	
25% Percentile	0.190	2.118	
Median	0.520	6.220	
75% Percentile	1.365	20.38	
Maximum	27.72	563.9	

For categorical variables (age group, sex, and race), the Chi-squared test or Fisher's exact test was used, as appropriate. For continuous variables (cAMP levels), linear regression models were used with age and sex as covariates across clinical groups. To meet the normalization assumption required for linear regression, cAMP levels were log-transformed. cAMP levels are presented as pmol per 60 μl serum. The analysis was performed using R version 4.4.1.

Supplementary Table 2. Demographics, lung functions (pre- and post-bronchodilator responses) and cAMP levels by asthma severity

Serum Donor (no. [%])	Nonsevere Asthma	Severe Asthma	*P* value
Age (yr, range)			0.7051
20-39	10/48 (21%)	8/39 (20%)	
40-59	21/48 (44%)	14/39 (36%)	
60+	17/48 (35%)	17/39 (44%)	
Sex			0.6761
Male	14/48 (29%)	13/39 (33%)	
Female	34/48 (71%)	26/39 (67%)	
Race			0.8436
White	35/48 (73%)	25/39 (64%)	
Black or African American	7/48 (15%)	6/39 (15%)	
Asian	4/48 (8%)	5/39 (13%)	
Hispanic	2/48 (4%)	1/39 (3%)	
Other / Missing	0/48 (0%)	2/39 (5%)	
**Serum Donor (Mean [SD])**			
Pre-bronchodilator, % pred.			
FEV_1	78.9 (17.8)	69.8 (22.7)	0.0394
FVC	91.7 (14.9)	84.7 (20.1)	0.0628
FEV_1/FVC	85.0 (9.7)	81.0 (13.7)	0.1198
Post-bronchodilator, % pred.			
FEV_1	87.4 (17.7)	75.9 (23.2)	0.0106
FVC	97.0 (14.0)	89.3 (20.4)	0.0407
FEV_1/FVC	89.1 (9.7)	83.8 (14.0)	0.9857
**Serum cAMP Levels (pmol)**			0.8985
Minimum	0.291	0.435	
25% Percentile	1.994	2.253	
Median	4.547	8.625	
75% Percentile	13.00	26.09	
Maximum	563.9	273.4	

Asthma severity was defined as “nonsevere” and “severe” according to European Respiratory Society / American Thoracic Society criteria.[11] For categorical variables (age group, sex, and race), the Chi-squared test or Fisher’s exact test was used. For continuous variables, the Student’s t-test (two-tailed) or linear regression models were used with adjusting for age and sex as covariates across clinical groups. The analysis was performed using R version 4.4.1. FEV_1, forced expired volume in 1 second; FVC, forced vital capacity.
